# FEMORAL NECK FRACTURES GARDEN I AND II: EVALUATION OF THE DEVIATION IN LATERAL VIEW

**DOI:** 10.1590/1413-785220172502169349

**Published:** 2017

**Authors:** Natália Zalc Leonhardt, Lucas da Ponte Melo, David Gonçalves Nordon, Fernando Brandão de Andrade e Silva, Kodi Edson Kojima, Jorge Santos Silva

**Affiliations:** 1 Universidade de São Paulo, Faculdade de Medicina, Hospital das Clínicas, Instituto de Ortopedia e Traumatologia, São Paulo, SP, Brazil.

**Keywords:** Femur neck, Radiography, Classification

## Abstract

**Objective:**

To evaluate the rate of deviation in the lateral radiographic incidence in patients with femoral neck fracture classified as non-diverted in the anteroposterior view (Garden I and II).

**Methods:**

Nineteen selected patients with femoral neck fractures classified as Garden I and II were retrospectively evaluated, estimating the degree of deviation in the lateral view.

**Results:**

Fifteen cases (79%) presented deviations in lateral view, with a mean of 18.6 degrees (±15.5).

**Conclusion:**

Most fractures of the femoral neck classified as Garden I and II present some degree of posterior deviation in the X-ray lateral view. ***Level of Evidence III, Retrospective Comparative Study.***

## RESUMO

**Objetivo:**

Avaliar a taxa de desvio na incidência radiográfica lateral em pacientes com fratura do colo femoral classificadas como não desviadas na incidência anteroposterior (Garden I e II).

**Métodos:**

foram avaliados retrospectivamente 19 pacientes selecionados com fraturas do colo do fêmur classificadas como Garden I e II, estimando-se o grau de desvio na incidência radiográfica lateral.

**Resultados:**

Quinze casos (79%) apresentaram desvio no perfil, com média de 18,6 (± 15,5).

**Conclusão:**

A maioria das fraturas do colo femoral classificadas como Garden I e II apresenta algum grau de desvio posterior na incidência radiográfica lateral. ***Nível de Evidência III, Estudo Retrospectivo Comparativo*** .

## INTRODUCTION

Femoral neck fractures are common in the elderly population, accounting for 50% of hip fractures.^1^ Frequently used classifications include the Arbeitsgemeinschaft fur Osteosynthesefragen/Orthopaedic Trauma Association (AO/OTA) scale, the classification by Pauwels,^2^ ^)^ and the Garden classification. The Garden classification^3^ ^)^ has significant clinical importance since it is frequently used in indicating treatment. It divides femoral neck fracture into 4 grades; types I (incomplete with impaction in valgus) and II (complete without displacement) are considered non-displaced, and types III (partial displacement) and IV (complete displacement) are considered displaced.

Although fractures are typically diagnosed with at least two radiographic views, recent studies question the need to perform profile x-ray imaging in proximal femur fracture.^4^ ^-^ ^8^ These studies have suggested algorithms^8^ in which lateral radiographs should only be requested when the fracture exhibits no displacement in the anteroposterior view, which occurs in 18% of femoral neck fractures, or when occult fractures are suspected.^9^

The average failure rate for displaced fractures of the hip in the elderly is 42% for internal fixation, 11% for partial arthroplasties, and 6% for total arthroplasties.^10^ ^-^ ^12^ Other authors, however, indicate that surgical outcome is influenced by the dorsal angle,^13^ ^-^ ^15^ ^)^ with posterior deviation of 20 degrees or more indicating re-operation.^14^ ^)^

The objectives of this study were to assess the rate of subjects with displacement in the lateral radiograph in patients with femoral neck fracture classified as not displaced in the anteroposterior view (Garden I and II); to measure the displacement in the lateral radiograph; and to investigate the association between the presence of displacement in the profile view and the occurrence of complications.

## METHODS

**Figure 1:**
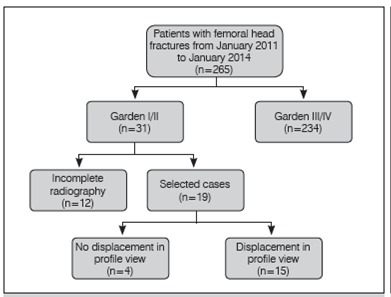
Flowchart showing study design.

A retrospective survey was conducted of all femoral neck fractures operated in our department from January 2011 to January 2014. From these we selected the cases classified as Garden type I and II to evaluate the anteroposterior and lateral x-rays. The study was approved by the local ethics committee (process number: 1.051.880). Cases where the x-rays were inadequate or the radiographic sequence was incomplete were excluded. Participants were not excluded because of age or multiple fractures. The flow of patients in the study is shown in Figure 1.

Radiography was performed in our institution according to the following standards:

*Anteroposterior view:* patient in supine position on the examination table with legs extended in neutral abduction and internal rotation of 15 degrees; anteroposterior X-ray was taken of the pelvis with the film centered on the pubic symphysis with the X-ray tube 120 cm from the table.

*Profile view (lateral):* patient supine, limb to be X-rayed extended and the opposite side semiflexed and abducted; hip profile was radiographed with the beam angled 45 degrees cranially.^16^

Demographic data, type of surgical intervention performed and outcome, as well as the need for reintervention were recorded. The x-rays were classified and measured by an evaluator specifically trained for this task. For the profile view, a normal cervico-diaphyseal angle of 180° was considered, and any anterior or posterior angulation was regarded as fracture displacement.

Descriptive statistics were compiled for the demographic data and clinical outcomes, and the association between the presence of displacement and the occurrence of complications was investigated using the chi-squared test. A sample was not calculated in advance, since the study's sample was defined by the number of patients who received surgery during the defined period. The statistical analysis was performed using Stata 13.0 software (StataCorp. 2013. Stata Statistical Software: Release 13. College Station, TX: StataCorp LP).

## RESULTS

Nineteen patient records were reviewed and included in the study. The demographic data for the patients included in the study are shown in Table 1. The fracture characteristics are shown in Table 2, including Pauwels classification and AO/OTA classification. Fifteen of the 19 cases slowed displacement in the profile view (79%), all in the posterior direction.

**Table 1:** Demographic data.

	N=19
Age	58.4 (± 22.3)
Sex	
Male	6 (32%)
Female	13 (68%)
Mechanism	
Hit by vehicle	2 (11%)
Motorcycle accident	2 (11%)
Automobile accident	2 (11%)
Spontaneous fracture	2 (11%)
Fall from standing height	11 (56%)

**Table 2:** Fracture characteristics.

	N = 19
Side	
Right	10 (53%)
Left	9 (47%)
Garden classification	
Garden I	6 (32%)
Garden II	13 (68%)
Pauwels Classification	
Pauwels A	3 (16%)
Pauwels B	10 (53%)
Pauwels C	6 (31%)
AO Classification	
31B1	9 (47%)
31B2	10 (53%)
31B3	0
Cervico-diaphyseal angle in the AP view	138.7º (± 16.7)
Displacement in the profile view	
No	4 (21%)
Yes	15 (79%)
Displacement angle in the profile view	18.6º (± 15.5)

Mean time to surgery was 7.3 days (±5.9). Fixation with three cannulated screws was used in 17 patients (89%), bipolar hemiarthroplasty was used in one (5%), and a cephalomedullary rod was used in one patient (5%).

Of the patients assessed, four (21%) had complications from the procedure and required re-operation. In all of these cases, the initial treatment used fixation with cannulated screws in an inverted triangle. The mean displacement in the profile view in this sub-group was 26°, and the average age was 56.2 years. There were no cases of failure in the cases where no displacement was seen in the profile view. The association between the presence of displacement in the profile view and the occurrence of complications was not statistically significant (p = 0.240). 

## DISCUSSION

Our study identified posterior displacement in 79% of the patients with femoral neck fracture classified as Garden grades I or II. Palm et al.^14^ defined 20 degrees of posterior deviation as an independent predictive factor for re-operation in a study that followed patients for one year. These authors reported a mean angle of 13 degrees, with 23% reoperations. In our study, four patients (26%) required re-operation. In the retrospective assessment, two of these patients had displacement of more than 20 degrees (35 and 41 degrees), while two had displacement of less than 20 degrees (14 and 18 degrees), confirming the findings of Palm et al.^14^

Lapidus et al.^17^ performed a study similar to Palm et al.,^14^ with a higher number of cases and a five-year follow-up period, and found 12% reoperations and mean posterior angulation of 12 degrees. The authors demonstrated association between the presence of posterior displacement and the need for re-operation, but found no correlation between the degree of displacement and reoperations.

Some studies recommend not performing lateral x-rays in cases of clear displacement, thus reducing patient exposure to radiation and costs. Almazedi et al.^4^ showed that adding a lateral x-ray to the anteroposterior view increases the sensitivity of diagnosis for displacement fractures from 53% to 91%, and specificity from 88% to 91% in proximal femur fractures. Riaz et al.^5^ confirmed these findings in a similar study. Both concluded that profile x-rays were effective in differentiating displacement fractures that did not appear to be displaced in the anteroposterior view, but they did not alter procedure in the cases in which the anteroposterior view showed a fracture with displacement.

Considering these findings, performing a lateral x-ray in femoral neck fractures that do not show displacement in the anteroposterior plane is relevant. Khan et al.^18^ demonstrated that the presence of posterior femoral neck multifragmentation in non-displaced and displaced fractures showed no association with complications, which occurred in 18% of non-displaced fractures. Parker et al.^19^ observed that the results of fixation in non-displaced fractures were better than arthroplasty in displaced fractures. However, the need for reintervention was greater in non-displaced fractures that were treated with fixation (17%). In another study, Paker et al.^20^ identified non-union in 30% of cases of displaced fractures treated with internal fixation, compared to 9% in fractures without displacement. Conn and Parker^13^ demonstrated that advanced age, associated with posterior angle and the need for walking assistance prior to the fracture are indicative of non-union after fracture of the proximal femur.

The gender distribution of our series was similar to that of other studies,^4^ ^-^ ^8^ ^,^ ^17^ but the average age was lower (58 years); this was explained by the inclusion of mostly young patients with multiple trauma. Despite the significant rate of patients with posterior displacement, fixation with cannulated was applied in most cases. Our re-operation rate of 21% demonstrates that the surgical method must consider the findings from the lateral x-ray. Computed tomography can be used in cases where the x-rays are inconclusive. It is important to emphasize the lack of a statistically significant association between posterior displacement and the re-operation rate, which may represent a false negative result due to the limited size of our sample.

This study has a number of limitations. The limited case series decreases the power to search for associations between the presence of displacement and complications. The retrospective design predisposes the study to selection bias and limited the registration of information, mainly related to patient follow-up.

## CONCLUSION

Most of the femoral neck fractures classified as Garden grades I and II exhibit some degree of posterior displacement in the lateral x-ray. This finding, coupled with the significant rate of synthesis material failure in these patients, should be considered in choosing the treatment method.
